# Immune Thrombocytopenia Successfully Controlled by Dissection of an Enlarged Mediastinal Lymph Node Metastasis from Squamous Cell Carcinoma of Unknown Primary: A Case Report

**DOI:** 10.70352/scrj.cr.25-0642

**Published:** 2026-01-16

**Authors:** Yoshihito Iijima, Takaki Mizoguchi, Masahito Ishikawa, Shun Iwai, Nozomu Motono, Hidetaka Uramoto

**Affiliations:** Department of Thoracic Surgery, Kanazawa Medical University, Ishikawa, Japan

**Keywords:** Immune thrombocytopenic purpura, surgery, nodal dissection, carcinoma of unknown primary

## Abstract

**INTRODUCTION:**

Immune thrombocytopenia (ITP) is an autoimmune hematologic disorder characterized by a reduced platelet count resulting from immune-mediated platelet destruction and/or impaired thrombopoiesis. This condition is often associated with malignant tumors, making perioperative management crucial to maintain hemostasis during and after surgery. Herein, we report a case of ITP successfully controlled following surgical dissection of a large mediastinal lymph node metastasis originating from squamous cell carcinoma of unknown primary.

**CASE PRESENTATION:**

A 57-year-old man with poorly controlled ITP was found to have progressively enlarging mediastinal lymph nodes on chest CT over 4 months. The largest lymph node measured 72× 37× 31 mm. The patient received preoperative intravenous immunoglobulin therapy (0.4 g/kg/day) for 4 days, after which mediastinal lymph node dissection was safely performed without hemorrhagic complications. The platelet count improved transiently after surgery. Histopathology revealed metastatic poorly differentiated squamous cell carcinoma, but imaging failed to identify a primary lesion, leading to a diagnosis of squamous cell carcinoma of unknown primary.

**CONCLUSIONS:**

With appropriate preoperative management, the platelet count was effectively controlled, allowing safe removal of the large mediastinal lymph node. As the platelet level improved postoperatively, prednisolone tapering was initiated. Given that recurrent malignancy may precipitate a decline in platelet count, close follow-up is warranted.

## Abbreviations

#2R
: upper paratracheal lymph nodes
#4R
: lower paratracheal lymph nodes
AV
: azygos vein
CUP
: carcinoma of unknown primary
FDG
: 2-deoxy-2-(^18^F)-fluorodeoxyglucose
ITP
: immune thrombocytopenia
IVIG
: intravenous immunoglobulin
LN
: lymph node
PSL
: prednisolone
SVC
: superior vena cava
VATS
: video-assisted thoracic surgery

## INTRODUCTION

ITP is an autoimmune hematologic disorder characterized by a reduced platelet count resulting from immune-mediated platelet destruction and/or impaired thrombopoiesis.^1,2)^ Although ITP is commonly recognized as a hematologic and immunologic complication of non-Hodgkin lymphoma, it can also rarely occur in association with solid malignant tumors other than lymphoma.^3,4)^ In patients with ITP, perioperative management is critical to maintain hemostasis during and after surgery, as thrombocytopenia significantly increases bleeding risk. Herein, we report a case in which surgical dissection of a large mediastinal LN metastasis from squamous cell CUP was successfully performed in a patient with ITP, resulting in improved platelet control following the procedure.

## CASE PRESENTATION

**Fig. 1:**
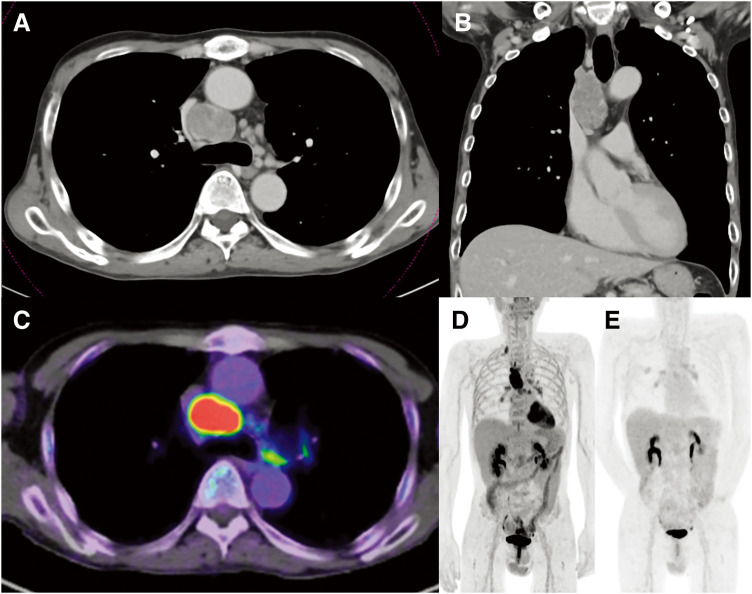
Preoperative findings. Chest CT revealed enlarged mediastinal LNs, measuring 72 × 37 × 31 mm in size. (**A**) Coronal view and (**B**) horizontal view. Preoperative PET-CT with FDG demonstrated tracer uptake (**C**) in the mediastinal LN and (**D**) faint accumulation in the bilateral hilar LN and the right subclavian LN. (**E**) Postoperative PET-CT performed 4 months after surgery showed no FDG accumulation suggestive of a primary tumor. FDG, 2-deoxy-2-(^18^F)-fluorodeoxyglucose; LN, lymph nodes

A 57-year-old man with a history of putaminal hemorrhage and previous endoscopic treatment for colon cancer was urgently admitted to the hospital with hematemesis and melena 6 months before referral to our department. Upper gastrointestinal endoscopy revealed a hemorrhagic gastric ulcer and thrombocytopenia. After ulcer treatment, *Helicobacter pylori* eradication therapy was initiated. Bone marrow aspiration showed numerous megakaryocytes, confirming a diagnosis of ITP. The patient was treated with PSL, fostamatinib, and eltrombopag; however, platelet levels remained unstable. Chest CT demonstrated progressively enlarging mediastinal LNs over 4 months (**Fig. 1A** and **1B**), the largest measuring 72 × 37 × 31 mm. At presentation to our department, the platelet count had decreased to 7000/μL despite continued administration of PSL 5 mg, fostamatinib 300 mg, and eltrombopag 50 mg daily. Laboratory evaluation revealed elevated soluble IL-2 receptor antibody at 582 IU/mL, carcinoembryonic antigen at 11.1 ng/mL, and squamous cell carcinoma antigen at 16.3 ng/mL. PET-CT using FDG demonstrated intense tracer uptake in the mediastinal LN (**Fig. 1C**) and faint uptake in the bilateral hilar and right subclavian LNs (**Fig. 1D**). On re-evaluation of the CT scan prior to surgery, the right subclavian LN had decreased in size, and no superficial LNs were palpable; therefore, right supraclavicular LN biopsy was not performed. A joint conference was held to discuss radiation therapy and the surgical removal of the mediastinal LNs. Given the potential of obtaining a definitive histological diagnosis and the possibility that complete resection would control ITP, the decision was made to proceed with surgical resection first. If intraoperative findings indicated that complete resection was not possible, or if surgery was completed with biopsy alone, radiation therapy would be performed. The patient agreed to this plan and provided informed consent.

**Fig. 2:**
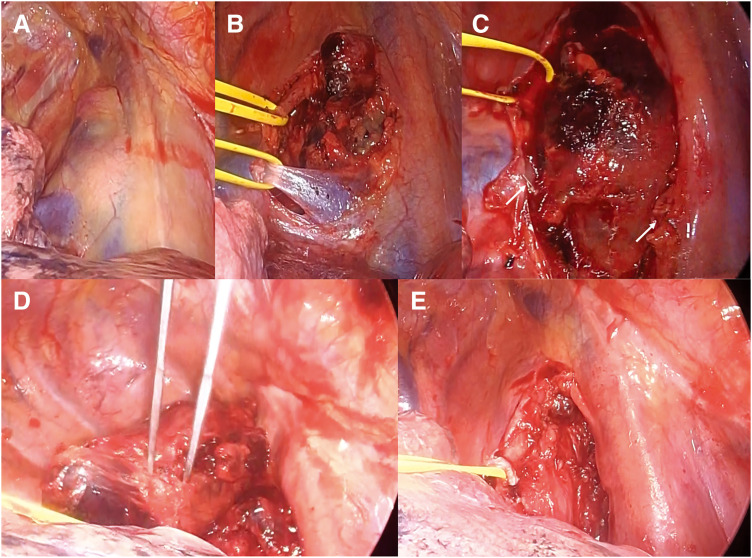
Intraoperative findings. (**A**) The enlarged LNs were identified on the dorsal side of the superior vena cava. (**B**) The vagus nerve and AV were exposed and taped, and (**C**) the AV was transected using an automatic stapling device. (**D**) The enlarged #4R was dissected and removed. (**E**) The remaining upper paratracheal LN, #4R, and LNs around the main bronchus were dissected. #4R, lower paratracheal lymph nodes; AV, azygos vein; LN, lymph nodes

After admission, the patient received intravenous IVIG therapy (0.4 g/kg/day) for 4 days, which increased the platelet count to 160000/μL on the day of surgery. Surgery was initiated using a 3-port complete VATS approach. LNs were identified on the dorsal side of the SVC (**Fig. 2A**). The vagus nerve and AV were exposed and preserved (**Fig. 2B**), and the #2R was excised for intraoperative frozen section analysis, which confirmed squamous cell carcinoma. Based on this finding, the procedure was converted to a hybrid VATS technique with an 8-cm mini-thoracotomy and 2 additional ports. The AV was transected using an automatic stapling device (**Fig. 2C**). The #4R, located free from major vascular and airway structures, was carefully dissected and removed while maintaining close attention to the SVC, pulmonary artery, trachea, and main bronchus (**Fig. 2D**). The remaining #2R, #4R, and peribronchial LNs were subsequently dissected (**Fig. 2E**). The total operative time was 118 min, with blood loss estimated at 30 mL. Although the platelet count was maintained at 140000/μL intraoperatively, platelet transfusion was administered as a preventive measure. On POD 1, the platelet count increased to 193000/μL, and the chest drain was removed without evidence of chyle leakage. By POD 4, the platelet count had decreased to 41000/μL; however, no bleeding tendency was observed.

**Fig. 3:**
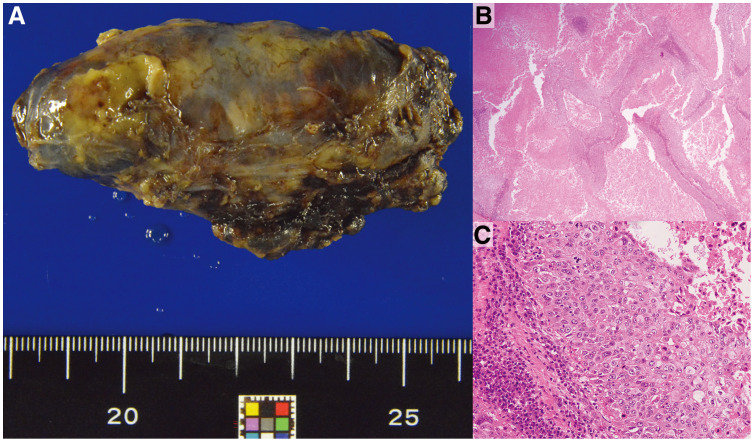
Pathological findings. (**A**) Macroscopically, the resected #4R was enlarged, measuring 80 mm in diameter. Histologically, atypical large cells with enlarged, irregular nuclei and acidophilic cytoplasm were proliferating in an irregular nest-like pattern, and the tumor was diagnosed as poorly differentiated squamous cell carcinoma. (**B**) H&E staining, ×4 magnification. (**C**) H&E staining, ×10 magnification. #4R, lower paratracheal lymph nodes; H&E, Hematoxylin and eosin; LN, lymph nodes

Macroscopically, the resected #4R was enlarged, measuring 80 mm in diameter (**Fig. 3A**). Histologically, atypical large cells with irregularly enlarged nuclei and acidophilic cytoplasm proliferated in an irregular, nest-like pattern, leading to a diagnosis of poorly differentiated squamous cell carcinoma (**Fig. 3B** and **3C**). Immunohistochemical analysis revealed that the atypical cells were positive for CK7 and CK5/6 in most areas, CK20 in a few areas, and GATA3 in a small subset. The cells were negative for p16, CD5, EBER, TTF-1, napsin A, synaptophysin, calretinin, GCDFP-15, PAX8, thyroglobulin, p40, p63, and SATB2, and no markers suggestive of a primary site were identified.

**Fig. 4:**
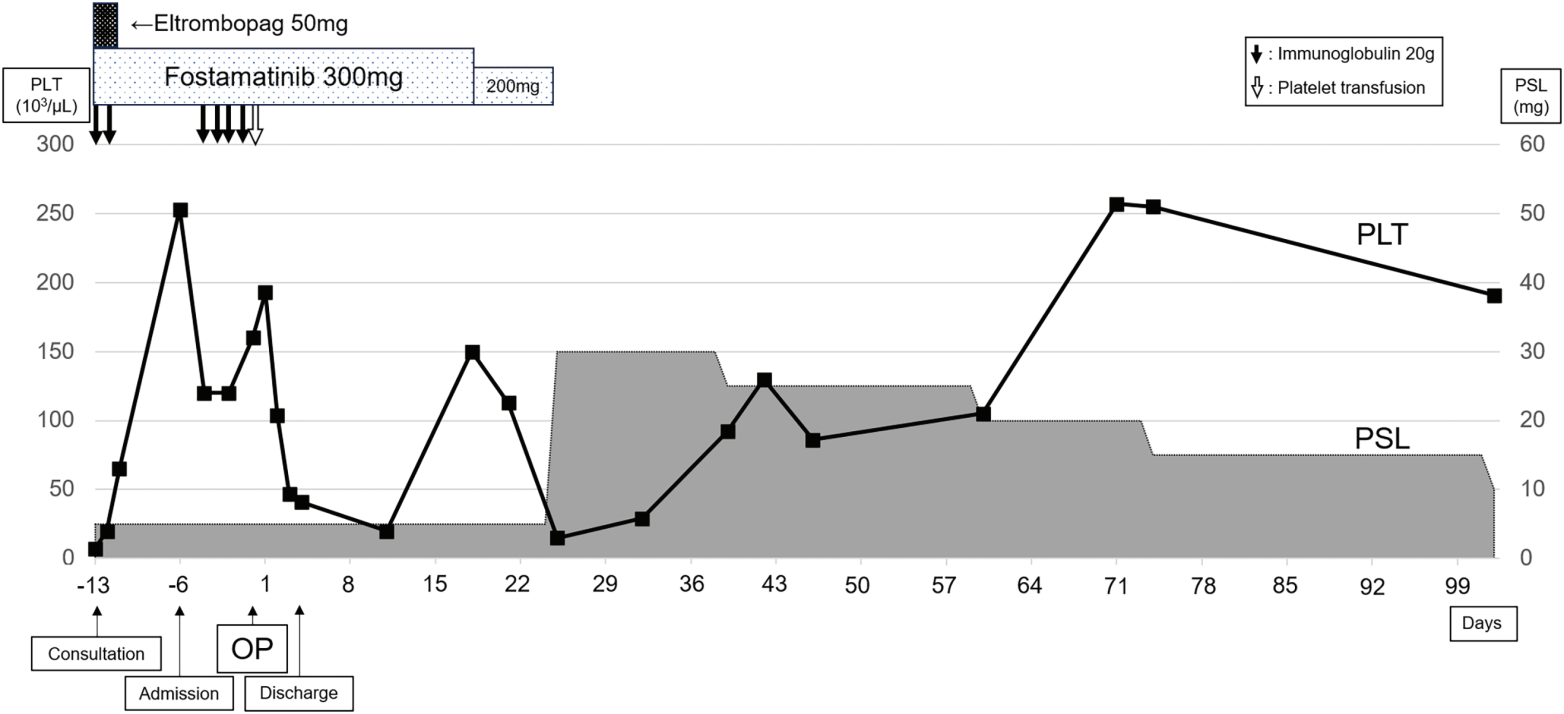
Perioperative clinical course. The PLT counts are shown by solid black lines, and the gray area graph shows the amount of PSL. On POD 25, fostamatinib was discontinued because of liver dysfunction, and PSL was increased from 5 to 30 mg. On POD 102, the PLT count remained at 191000/μL, and PSL was tapered to 10 mg. OP, operation; PLT, platelet; PSL, prednisolone

The perioperative clinical course is illustrated in **Fig. 4**. Following discharge, the platelet count initially showed transient improvement but declined again when the fostamatinib dose was reduced to 200 mg because of liver dysfunction. On POD 25, fostamatinib was discontinued for worsening hepatic function, and the PSL dose was increased to 30 mg. As platelet levels recovered, PSL was gradually tapered to 10 mg. By POD 102, the platelet count remained stable at 191000/μL. PET-CT performed 4 months after surgery to evaluate for a primary tumor showed disappearance of tracer uptake in the right supraclavicular LNs, with only reactive uptake in the bilateral hilar nodes (**Fig. 1E**). Eight months postoperatively, no recurrence was detected, the platelet count remained stable, and PSL therapy was successfully discontinued.

## DISCUSSION

ITP is a representative autoimmune phenomenon frequently associated with lymphomas. It may also occur as a rare paraneoplastic syndrome in various solid malignancies, including lung, breast, ovarian, renal, and prostate cancers.^3,5)^ Krauth et al. analyzed 68 reported cases of ITP associated with solid tumors and found that the condition most commonly occurred in patients with lung and breast cancers.^3)^ To the best of our knowledge, this is the first reported case of ITP associated with mediastinal LN metastasis from CUP. Miyoshi et al. analyzed 70 cases of hilar and mediastinal LN metastasis from CUP in Japan and reported that 81% of patients were male. The predominant histologic type was adenocarcinoma (41%), followed by small cell carcinoma (19%), large cell carcinoma (17%), and squamous cell carcinoma (16%).^6)^ Additionally, 43% of these cases involved isolated mediastinal LN disease.^6)^

When evaluating the resectability of mediastinal LNs, both nodal size (specifically, whether “bulky”) and infiltration into adjacent structures are critical considerations.^7,8)^ Although the definition of bulky LN remains controversial, international guidelines define “non-bulky” nodes as those that are easily measurable, with a short-axis diameter less than 3 cm, and free from invasion of major mediastinal structures, including the trachea and great vessels.^7,8)^ In the present case, PET-CT findings initially suggested that the lesion might correspond to N3 lung cancer. However, FDG uptake in the paratracheal LN was significantly more intense and heterogeneous than in the bilateral hilar and right supraclavicular LNs, and the right subclavian LN had decreased in size. Furthermore, the upper mediastinal LN did not invade surrounding major structures, such as the great vessels or trachea. Based on these findings, we concluded that complete resection could be achieved through mediastinal LN dissection. Therefore, upper mediastinal LN dissection was performed using a hybrid VATS approach with an 8-cm mini-thoracotomy and 2 ports. Even in cases with bulky LNs, safe and complete resection without blind spots was feasible with this approach.

There are numerous major blood vessels in the thoracic cavity; therefore, meticulous preparation is essential in thoracic surgery, as vascular injury can result in catastrophic outcomes.^9)^ In adult patients undergoing major non-cardiovascular or non-neuroaxial procedures, platelet transfusion is recommended when the platelet count is less than 50000/μL.^10)^ Accordingly, in patients with ITP requiring surgery, preoperative management should aim to maintain platelet counts at or above 50000/μL. Spahr et al. reported that platelet transfusion in combination with high-dose IVIG therapy resulted in a rapid and sufficient rise in platelet counts.^11)^ Forty ITP patients (mean platelet count, 10000/µL) who were actively bleeding required anticoagulation or were scheduled for surgery and received 1.0 g/kg of IVIG as a 24-h continuous infusion, together with 1 apheresis platelet dose every 8 h (38 patients [95%] also received steroids). Average platelet counts at 24 and 48 h increased to 55000 and 69000/µL, respectively. Initial bleeding was controlled in all patients, and no perioperative hemorrhagic complications occurred. Similarly, Watanabe et al. reported a successful case of sleeve middle and lower lobectomy for lung cancer following preoperative IVIG therapy.^5)^ These findings suggest that platelet transfusion for ITP should be considered alongside appropriate management of the underlying disease. In the present case, IVIG therapy was administered for 4 days before surgery, increasing the platelet count to 160000/μL immediately before the operation. Although the platelet count decreased to 47000/μL on POD 3, no rebleeding occurred, and the postoperative course was uneventful. Nevertheless, careful follow-up is warranted because ITP may worsen in association with cancer recurrence.

## CONCLUSIONS

With appropriate preoperative management, platelet levels were effectively controlled, allowing safe resection of the large mediastinal LN. As the platelet count recovered, PSL was gradually tapered. If CUP recurs, a subsequent decline in platelet count is anticipated; therefore, close hematologic surveillance is essential.

## DECLARATIONS

### Availability of data and materials

All data generated or analyzed during this study are included in this published article.
